# Oil exposure alters social group cohesion in fish

**DOI:** 10.1038/s41598-019-49994-1

**Published:** 2019-09-18

**Authors:** Tiffany Armstrong, Alexis J. Khursigara, Shaun S. Killen, Hannah Fearnley, Kevin J. Parsons, Andrew J. Esbaugh

**Affiliations:** 1 University of Glasgow, Institute of Biodiversity, Animal Health and Comparative Medicine, Glasgow, G12 8QQ UK; 2 University of Texas at Austin, Marine Science Institute, Port Aransas, Texas 78373 USA

**Keywords:** Environmental impact, Marine biology

## Abstract

Many animal taxa live in groups to increase foraging and reproductive success and aid in predator avoidance. For fish, a large proportion of species spend all or part of their lives in groups, with group coordination playing an important role in the emergent benefits of group-living. Group cohesion can be altered by an array of factors, including exposure to toxic environmental contaminants. Oil spills are one of the most serious forms of pollution in aquatic systems, and while a range of effects of acute oil exposure on animal physiology have been demonstrated, sub-lethal effects on animal behavior are relatively under-studied. Here we used an open-field behavioral assay to explore influence of acute oil exposure on social behavior in a gregarious fish native to the Gulf of Mexico, Atlantic croaker (*Micropogonias undulatus*). We used two oil concentrations (0.7% and 2% oil dilution, or 6.0 ± 0.9 and 32.9 ± 5.9 μg l^−1^ ΣPAH_50_ respectively) and assays were performed when all members of a group were exposed, when only one member was exposed, and when no individuals were exposed. Shoal cohesion, as assessed via mean neighbor distance, showed significant impairment following acute exposure to 2% oil. Fish in oil-exposed groups also showed reduced voluntary movement speed. Importantly, overall group cohesion was disrupted when even one fish within a shoal was exposed to 2% oil, and the behavior of unexposed in mixed groups, in terms of movement speed and proximity to the arena wall, was affected by the presence of these exposed fish. These results demonstrate that oil exposure can have adverse effects on fish behavior that may lead to reduced ecological success.

## Introduction

Animals living in groups often must coordinate movements with group mates in space or time, with varying degrees of synchronicity^1,2^. This type of social behavior is particularly important, as group living may be beneficial for predator avoidance, foraging, and reproductive success^3,4^. This is especially true for fish, in which a large range of species spend some or all of their life within a group-living scenario^5,6^. Additionally, group movements allow individuals to reduce the energetic costs of swimming by taking advantage of vortices produced by groupmates^7–9^. In a shoal of fish, the series of individual decisions that result in cohesive collective behaviors are influenced by a range of abiotic and biotic factors, including food availability, water flow rate^7^, turbidity^10^, predator abundance^11^, and a suite of social cues^12^. In addition, social behaviors in fish are affected by various forms of anthropogenic environmental disturbance, including human-induced hypoxic episodes^13,14^, ocean acidification^15^, and anthropogenic noise^16^. Environmental contaminants such as industrial pollutants and heavy metals can also adversely impact fish social behavior, such as schooling and courtship^17,18^. This is particularly relevant as group decision making, social learning, and group responses to potential threats^19,20^ are all dependent on the behaviors of individual group members, with key individuals often influencing the behavior of an entire group^21,22^.

One of the most serious forms of anthropogenic disturbances is pollution from the release of petroleum products into aquatic environments^23,24^. Uncontrolled oil spills are the most well known form of crude oil pollution, which was epitomized by the *Deepwater Horizon* spill that released over 700 million L of crude oil into the Gulf of Mexico over a period of 84 days^25,26^. However, oil is also routinely released into aquatic habitats via shipping, terrestrial runoff, and dumping. Oil toxicity is driven by a class of chemicals known as polycyclic aromatic hydrocarbons (PAHs), and marine fish are particularly susceptible to these chemicals. Embryonic exposure can result in craniofacial and cardiac deformities that have been linked to mortality at very low concentrations (μg/L)^27–31^. In juvenile and adult life stages, acute oil exposure impairs cardiac performance^32–36^, which leads to reductions in swimming performance, maximum metabolic rate and aerobic scope^37–40^. Similarly, acute oil exposure can also cause changes in routine metabolism^41,42^. Importantly, the sub-lethal effects of an acute exposure event lasting only 24 hours can persist in the animal for multiple weeks^39,40^, which raises concern about the long-term ecological performance of exposed individuals.

Despite well-known physiological effects of oil pollution, there is surprisingly little known about the negative impacts of oil exposure on fish social behaviors. Behavioral characteristics are crucial when extending organismal toxicology to ecologically relevant population-level effects, as described in the adverse outcomes pathway framework^43^. Sociability, or an animal’s tendency to interact with conspecifics^44^, plays an important role in shoal behaviors. Shoals comprised of more social individuals have higher shoal cohesion, though exhibit a reduction in average swim speed and social alignment^45^. Further, individuals with lower sociability are more likely to swim faster and act as a leader within a school, with these individuals effectively initiating group movements^45^. Sociability is in turn influenced by individual metabolism with less social animals having higher standard metabolic rates^46^. Therefore, the previous mentioned negative impacts of oil exposure on metabolism^37–40^ and cardiac performance^32–34^, could imply a direct pathway for oil exposure to influence shoal cohesion. This was highlighted recently in a study of coral reef fish following acute oil exposure, whereby exposed individuals showed a suite of behavioral changes – habitat usage, thigmotaxis, and basic aspects of shoaling behavior – that significantly increased predation rates^47^. Further, sensory abilities, such as vision^48,49^, olfaction^50^, and input from the lateral line^51^, influence shoal cohesion, grouping choices, and coordinated movements. Any change in sociability, sensory ability, or locomotor capacity in any or all group members due to oil exposure could therefore disrupt overall group function with important ecological consequences.

To this end, the current study examined the effects of environmentally relevant levels of crude oil exposure on social behavior and group cohesion in Atlantic croaker (*Micropogonias undulatus*). This gregarious fish species is prevalent in the Gulf of Mexico and depends on estuarine environments, which can be particularly impacted by oil pollution. Specifically, we aimed to answer the following questions: 1) under acute oil exposure is shoal cohesion, or individual exploratory behaviors, different in an open-field when compared to non-exposed groups? and, 2) if a single individual is acutely exposed is the cohesion of the shoal, or the exploratory behaviors of other individuals altered?

## Results

### Oil chemistry analysis

**Figure 1:**
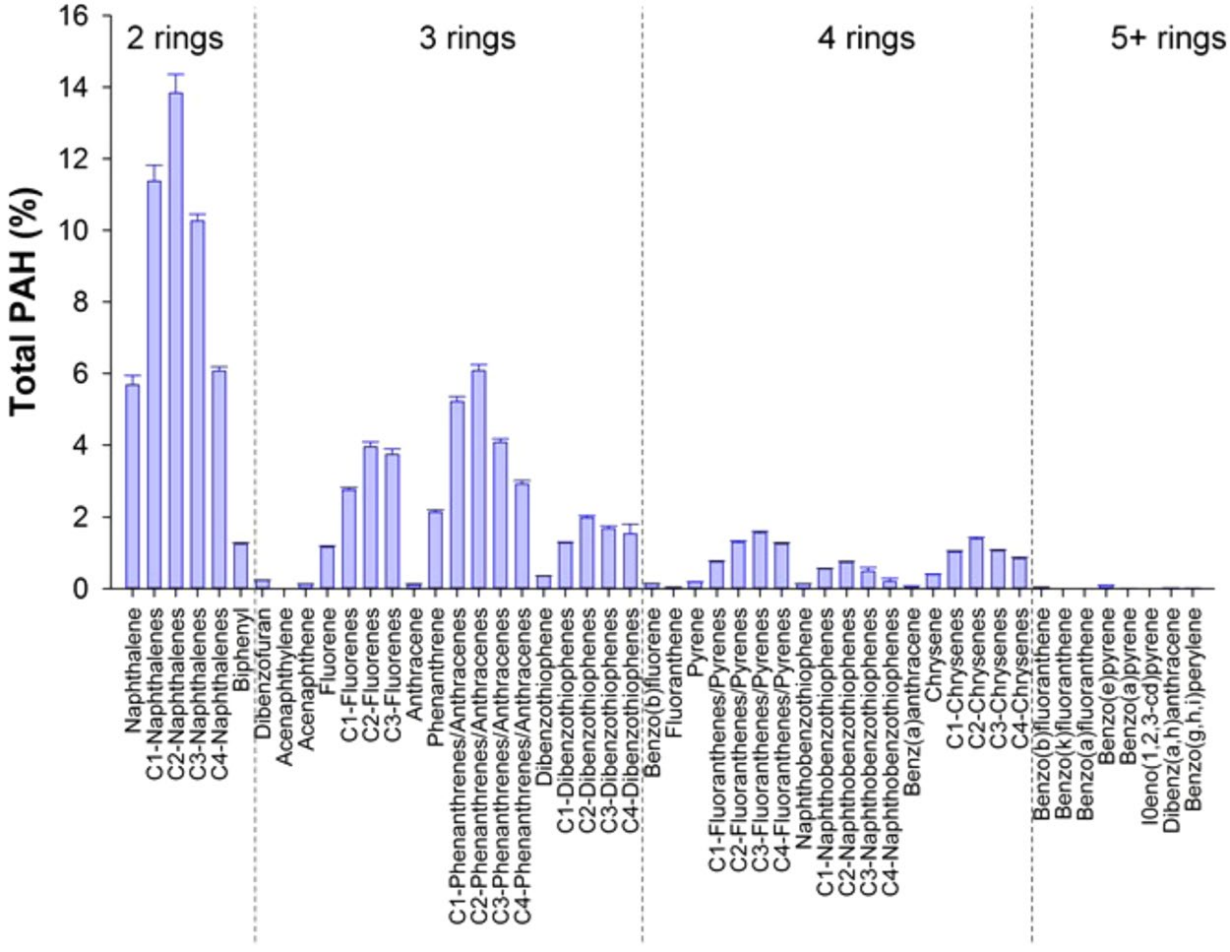
Relative composition of polycyclic aromatic hydrocarbons in the low and high concentration HEWAFs. 50 individual PAHs were measured and are shown on the X-axis. Dashed lines denote subclasses of PAHs.

HEWAFS from both the low and high concentrations were analyzed for 50 individual PAHs. As expected, for both HEWAFs, 2 ring PAHs were highest in abundance (47% and 53% in the low and high concentrations respectively) followed by 3 ring PAHs (41% and 36%), with the remaining being 4 and 5 ring PAHs (Fig. 1). Using initial and final concentrations, the average geometric mean (±SEM) for the low concentration was 6.0 ± 0.9 μg l^−1^ ΣPAH_50_ and for the high concentration was 32.9 ± 5.9 μg l^−1^ ΣPAH_50_.

### Effects of oil on group behavior

**Figure 2:**
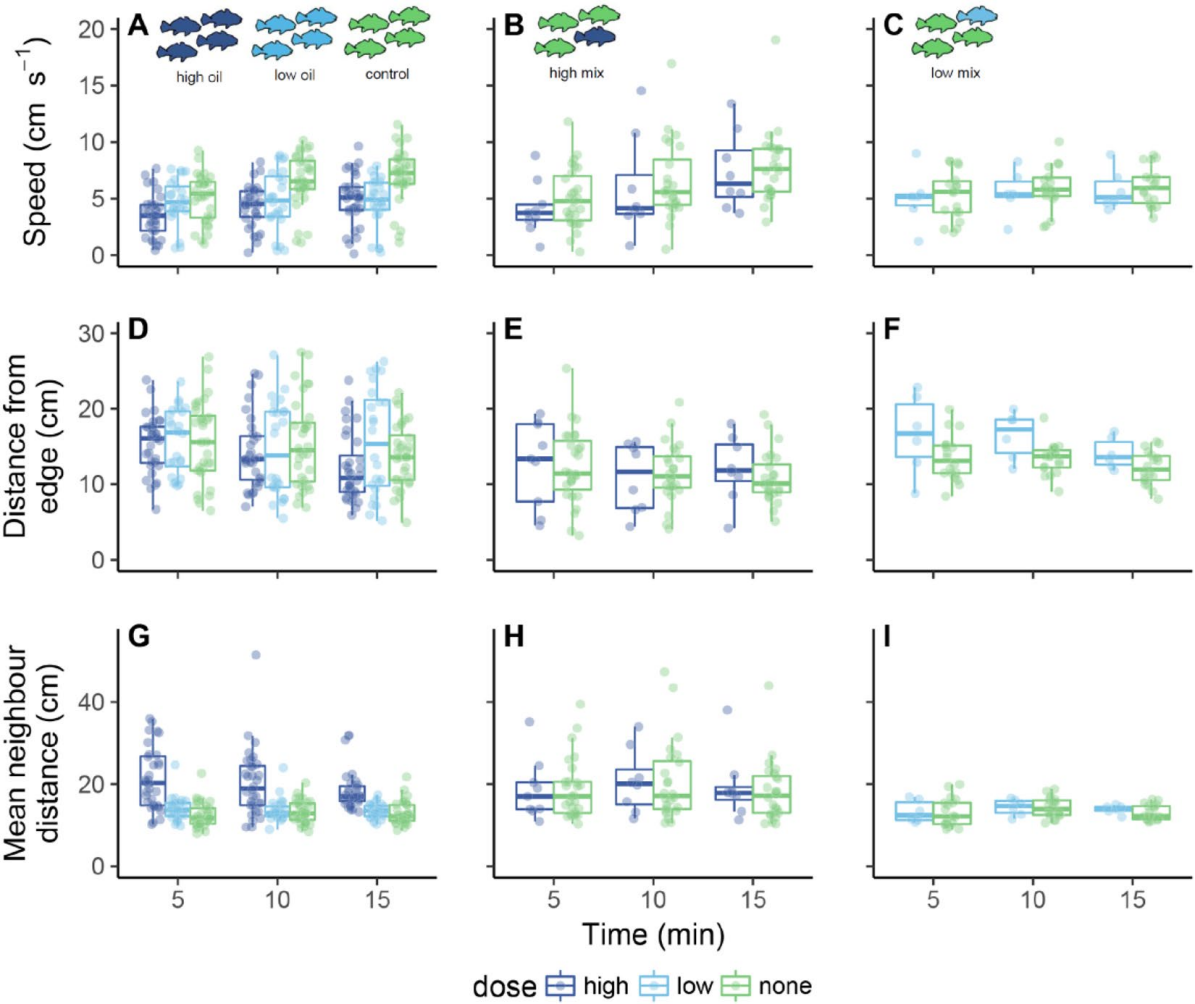
Responses to high (32.9 ± 5.9 μg l^−1^ ΣPAH_50_) or low (6.0 ± 0.8 μg l^−1^ ΣPAH_50_) oil exposure in juvenile Atlantic croaker in groups of four individuals swimming within an open field (n = 15 groups in total). Groups were composed of either control fish (that were not oil exposed; green); fish exposed to a high concentration of oil (dark blue); fish exposed to a low concentration of oil (light blue); three fish not exposed to oil plus one fish exposed to a high concentration of oil; or three fish not exposed to oil plus one fish exposed to a low concentration of oil. Each data point overlaid on the boxplots represents one fish within a group. Refer to Supplemental Tables 1, 2 and 3 for statistical comparisons among treatments.

While fish tended to increase their movement speed over the course of each 15 min trial, individuals in HO groups showed decreased speed of movement as compared to fish in all other treatments (Fig. 2a). Fish in HM groups were closer to the arena wall when compared to all other treatment groups (Table 1). Fish in HO and HM groups showed increased mean neighbor distances (Fig. 2g; Table 1). Within a given treatment there was generally large among-group variation for all behavioral indices, and overall, values for model R^2^_C_ was higher than R^2^_M_ (Tables 1 and S1).

**Table 1:** Results of a linear mixed effects models examining the factors influencing behavior in groups of fish receiving various levels of oil exposure.

	Estimate	SEM	df	t	p	R^2^_m_	R^2^_c_
**Speed (cm s** ^**−1**^ **)**							
intercept	1.65	0.999	146.54	1.653	0.101	0.2	0.832
mass	0.133	0.04	141.97	3.302	0.001		
time	0.153	0.013	289.13	11.947	<0.0001		
treatment							
high oil	−1.719	0.556	141.73	−3.093	0.002		
low oil	−1.115	0.606	141.74	−1.841	0.068		
high mix	0.078	0.539	143.59	0.145	0.885		
low mix	−0.397	0.599	141.73	−0.663	0.508		
**Distance to arena edge (cm)**							
intercept	16.319	1.899	148.7	8.592	<0.0001	0.085	0.721
mass	0.011	0.076	141.51	0.144	0.886		
time	−0.167	0.03	289.57	−5.482	<0.0001		
treatment							
high oil	−1.041	1.053	141.19	−0.989	0.325		
low oil	0.461	1.147	141.19	0.402	0.688		
high mix	−3.14	1.023	143.71	−3.069	0.003		
low mix	−1.242	1.133	141.18	−1.096	0.275		
**Mean distance to neighbors (cm)**							
intercept	1.078	0.048	151.7	22.453	<0.0001	0.283	0.78
mass	0.001	0.002	141.6	0.634	0.527		
time	−0.001	0.001	290.5	−0.617	0.538		
treatment							
high oil	0.019	0.026	141.2	7.052	<0.0001		
low oil	0.035	0.003	141.2	1.218	0.225		
high mix	0.152	0.026	144.3	5.908	<0.0001		
low mix	0.027	0.029	141.2	0.952	0.343		

For the fixed effect of “treatment”, control groups, which received no oil exposure, are the reference level. Models included individual nested within group as a random effect.

### Effects of oil-exposed individuals on non-exposed individuals within the same group

Within LM groups, treated individuals tended to be further from the arena walls than untreated fish (Fig. 2f; Tables S2 and S3). While there were no differences in distance from the wall between treated and untreated individuals within the HM groups, this is likely because even untreated fish within high mixed groups were closer to the arena walls than fish in control groups (Table S2). Untreated fish in HM groups showed increased mean neighbor distances compared to fish in control groups comprised entirely of unexposed fish (Fig. 2h; Tables S2 and S3).

## Discussion

The results here suggest that environmentally relevant oil exposure scenarios cause decreased cohesion in fish social groups. At the highest level of exposure, groups comprised of oil-exposed fish had increased distances between neighboring fish within groups. This effect was also observed in groups that contained a single high oil-exposed fish in a shoal of four, whereby untreated fish within the same group showed disruptions to normal behavior and greater distances between neighbors. Overall, these results indicate that exposure to oil pollution in aquatic environments has the potential to negatively affect fish social behaviors and group functioning.

The data presented here clearly demonstrate that an acute oil exposure of 32.9 ± 5.9 µg l^−1^ ΣPAH_50_ results in a less cohesive shoal and alters overall group behavior. While acute oil exposure has a well-documented suite of sub-lethal effects on fishes, the severity of sub-lethal endpoints on overall ecological performance can be difficult to ascertain. The concept of ecological death is useful in this context, as it draws an equivalency between immediate mortality and any toxicological impairment that reduces the ability to perform in the environment and produce offspring^17^. Shoaling for fish is a particularly noteworthy behavior in this regard as it has been shown to have positive effects in the context of foraging and predator avoidance^52^. For example, during predator attacks group cohesiveness allows groupmates to react to danger even when they themselves did not observe the attack^19^, and coordinated movements of prey fish during predator-prey interactions can serve to confuse predators. Any reductions in shoal cohesion and coordination will preclude individuals from experiencing the benefits of group living, and therefore put the individuals at greater risk.

The overall reduced speed of movement among groups of oil exposed fish could also reduce foraging ranges and the likelihood of encountering food while exploring a given environment^53^. Fish in swimming schools can also position themselves relative to groupmates such that they can reduce their own costs of movement by taking advantage of the vortices produced by other fish within the school^8,9,54^. Individual fish also prefer specific spatial positions within schools (e.g. front versus back, edge versus center) in relation to their own physiological and behavioral traits^55,56^, possibly contributing to the establishment of leader-follower dynamics and formation of characteristic social networks^57^. Reduced group cohesion may impact the tendency of fish to occupy their preferred or optimal positions within moving groups; however, further work is needed to examine the extent to which oil exposure may impair the ability of individual fish to occupy their preferred spatial position within social groups.

Individual speed of movement is also a determinant of leadership during directed group behaviors, with individuals being attracted to more active conspecifics and those that display directional, linear movements^16,58,59^. Reduced spontaneous activity therefore suggests that leadership capacity could be compromised in oil-exposed fish. Interestingly, however, exposed and unexposed fish in the HM groups all displayed decreased distances from the arena wall as compared to fish in other treatment groups. Although their ability to lead may be diminished, it is possible that exposed fish in HM groups were nonetheless altering the behavior of unexposed groupmates in an emergent manner that is not possible when all fish in a group are oil-exposed. A tendency to remain closer to the wall in an open field test is generally interpreted as a reduction in boldness or risk-taking behavior^60^ and it is possible that abnormal behavior in oil exposed fish elicited increased timidity in unexposed groupmates. This is the opposite effect to previous observations in larval red drum and coral reef species, both of which demonstrated an decrease in anxiety-like behavior individually^61^ and in groups^47^. Clearly, additional work is needed to investigate interactions between leader-follower dynamics and resultant levels of risk experienced by groupmates among fish with varying levels of oil exposure within the same social group.

It is notable that the negative effects on shoal cohesion occurred when even a single individual within the group was exposed to oil. It is important to remember that oil spills are heterogeneous events, and that the physiological effects of acute 24 h exposures have been shown to persist over prolonged time scales^39,40^. As such, it is plausible that in the wild, fish with varying levels of oil exposure will interact as they migrate or otherwise move within their environment. The results here demonstrate that in smaller shoals, the presence of a minority of exposed individuals can disrupt the behavior of the entire group. In other contexts, it has been observed that the presence of key individuals within a group can have a beneficial effect on group function^21^. For example, social learning can allow naïve groupmates to more quickly gain knowledge of foraging patches or danger from more informed individuals^20,58^. The ecological relevance of the influence of oil-exposed fish on the unexposed groupmates warrants further study, but this appears to be an example whereby one individual has a disproportionate effect on the behavior of the entire group. It has previously been speculated that individuals with particular behavioral or physiological traits may act as “keystone” individuals that have a disproportionate effect on the behavior or success of entire groups^21,57,62^. The current results also suggest that individual sensitivity or exposure to pollutants, and other adverse environmental conditions, could induce similar effects on social groups stemming from disrupted behavior in a minority of individuals.

In the current study, groups were relatively small, and so the particular behavioral tendencies of one individual would likely have a large influence on overall group behavior, even in groups of fish receiving equal oil exposures (and including the control groups). It is notable that for mean neighbor distance, groups of fish receiving high oil exposures (for all fish or one fish within the group) showed greatly increased among-group variation compared to all other group types. Additional research is required to understand how the effects observed in this study scale up to larger group sizes, and whether larger groups have reduced among-group variation in the behavior they display. In addition, fish social groups often display among-group assortment based on various morphological and possibly physiological characteristics^14,63^. An interesting area for future work would be to determine how groups of fish with varying phenotypic composition may show differential group-level responses to oil exposure.

The underlying cause for the oil induced behavioral changes is not immediately clear. The established paradigm for oil toxicity in fish is that sensitivity is driven by cardiac malformations caused by 3 ringed PAHs^64,65^. It is possible that the observed changes stem from altered metabolic characteristics, as a recent study demonstrated that similar oil exposure scenarios significantly reduced maximum metabolic rate and aerobic scope in this species^66^. Prior work has demonstrated that individuals with a higher metabolic rate can be less social, presumably because they prioritize food acquisition over the safety of being in a group^14^; however, such observations have not been extended to aerobic scope. It seems more likely that the mechanism relates to neurological or sensory impairment, both of which have recently been shown to be impacted in yolk-sac larval fish during developmental exposure^67,68^. This hypothesis was previously posited to explain the changes in anti-predator behavior observed in coral reef fish species^47^, and the reduced prevalence of thigmotaxis (i.e. anxiety behavior) in larval red drum^61^. Such developmental effects could impact the ability to detect and respond to shoal-inducing sensory stimuli, or the tendency to generate such stimuli.

In our experimental protocol, we ran control groups first during each experimental day to eliminate risk of oil contamination between trials. It is therefore conceivable that temporal variation in groups or trial order may have contributed to the observed differences among treatments. Several lines of evidence, however, suggest that if such effects occurred they are small relative to the direct effects of oil exposure. Firstly, the direction of temporal effects on a given behavioral measure (e.g. movement speed, which increased with time in the control group) was opposite the effects of the oil exposure treatments. This suggests that even if there were temporal effects on behavior it would have only served to reduce the magnitude of the observed effects from oil exposure. Furthermore, the HO and LO groups were run at the same time of day and yet still displayed differences in behavior. Individuals in LO groups behaved more similarly to control groups than those in the HO exposed groups, indicating that changes observed in the HO treatment were the result of the exposure level and not time of day or trial order. Additionally, the total time for all trials to be completed was relatively short (e.g. within 2 hours), reducing the potential of a temporal or hunger effect on behavior. It’s also important to note that in the HM groups, the unexposed individuals behaved differently than the oil-exposed fish within their group, again indicating that exposure level and not time of day or trial order was responsible for the observed changes in behavior.

In summary, this study demonstrates that oil exposure alters social cohesion in sub-adult Atlantic croaker. Indeed, exposure of a single individual can alter the behavior of the group. Importantly, the exposure scenarios used here are comparable to ΣPAH_50_ concentrations found in the Gulf of Mexico following the *Deepwater Horizon* spill^69^. Social behaviors are key to foraging, predator-avoidance, migration, and reproduction in most fish species, and so reduction in shoal cohesion is likely to have a range of adverse effects on these aspects of species’ ecology. Although additional work is required to more fully ascertain the mechanisms by which oil exposure alters behavior, the data presented here provide added support for the recently described phenomenon of oil induced behavioral impairments in fish^47^, while also highlighting novel effects of ecological significance. Future examinations of individual variation in sensitivity to oil exposure in a social context would provide further understanding of the selective effects of oil pollution on fish populations, and the potential implications for evolutionary trajectories^70,71^. Within-generation effects of pollution on behavioral plasticity, and indeed any anthropogenic stressor, are likely to have consequences for important ecological phenomena such as fish migrations, spawning aggregations, and survival during the juvenile stages, in which fish frequently use shoaling as an anti-predator strategy.

## Methods

### Oil preparation

To test the effects of oil exposure, oil was prepared following previously described standard protocols for high-energy water accommodated fractions (HEWAF)^28,30^. Briefly, non-weathered oil collected from the source of a Massachusetts pipeline, an appropriate surrogate for *Deepwater Horizon* source oil, was loaded with seawater (35ppt) at a rate of 1 g per l. Oil was blended in a heavy-duty blender (Waring Commercial, Connecticut, USA) at a low setting for 30 s, and then placed into a Teflon 1 L sieve funnel for 60 minutes. The lower 85% of the WAF was removed and used to generate oil exposures. All HEWAFs were prepared fresh for exposures. Oil was delivered under proper chain of custody and was stored at 4 °C until for roughly a year before used.

### Fish and exposure

To determine the effect of oil exposure on social behaviors we obtained 200 juvenile Atlantic croaker (9.8–15.1 cm standard length) from a commercial supplier. Fish were acquired in groups of 50 but were randomly split into smaller groups of 25 for acclimation. During acclimation, fish were held in 76 cm × 99 cm tanks, filled to 350L, at 24 ± 1 °C, and maintained with sterilized seawater (35 ppt) for two weeks before experiments began. Holding tanks were large enough to maintain water quality for groups of 25 or less. To maintain fish at conditions that were consistent to their native environment in the Gulf of Mexico, filtered seawater was piped directly from the Gulf into the lab.

Twenty-four h prior to exposure, 12 fish were randomly selected from each group, anaesthetized with 250 mg l^−1^ of MS222 (buffered with 500 mg l^−1^ NaHCO_3_), weighed, measured for total and standard length, and fitted with either a yellow, orange, green or purple 7 × 6 mm plastic bead for identification purposes. The 12 fish were divided into groups of four, which would be used for the behavioral trials. Selection for groups was random though there was an effort to group together fish with a similar body mass (±9.0 g). Fish were then allowed to recover (observed for return to equilibrium) for one h in an aerated opaque tank and returned to the original holding tank where they were food-deprived for 24 h prior to oil exposure. The following day the tagged fish were moved to individual aerated, 6.14 L tanks (24 × 16 × 16 cm), which were filled with fresh seawater, and exposed to one of three nominal concentrations of HEWAF (0, 0.7 and 2%) for 24 h. Concentrations were chosen based on levels recorded shortly after the *Deepwater Horizon* spill in 2010^69,72^, and have been used previously on this species^66^.

The behaviors of the following group compositions were tested: control (*N* = 9), low-oil exposure (LO; *N* = 7), high-oil exposure (HO; *N* = 8), low-mixed exposure (LM; *N* = 7) and high-mixed exposure (LM; *N* = 9). The mixed groups contained three control fish and one exposed fish of the respective oil dose. Each individual within the group had a unique colored bead, to allow for individual tracking and identification. A group of four fish was used to allow for the tracking of individuals within the group while also allowing all fish to move freely within the arena without constrained movement.

During the oil exposure protocol, standard water quality parameters were monitored, and seawater samples were taken for PAH analysis at the beginning and end of three exposures for each oil dose. Samples were taken throughout the course of the study. PAH analysis was performed commercially by ALS environmental under extraction protocol EPA 3510 C and measurement protocol 8270D SIM. Samples were spiked with fluorine-d10, fluoranthene-d10 and terphenyl-d14 to assess extraction efficiency, with general recovery of >80%, >90% and >90%, respectively. Detection limits ranged from 4.5–20.5 ng l^−1^ depending on the specific PAH. All samples were stored at 4 °C and delivered within one week of collection under proper chain of custody.

### Behavioral assays

To determine if exposure to oil influenced the exploration or group interactions of Atlantic croaker, the activity of groups of four fish were monitored in open-field tests. The arena for the open field consisted of a circular solid plastic tank with a diameter of 91.5 cm and filled with 7 cm of fresh UV sterilized seawater from the same source used to fill holding tanks. To visually contrast between the fish and the background, a white vinyl covering was used to line the arena. The fish were all caught with a dip net from the exposure tank, released into a 3 L container, where they were rinsed with fresh seawater, and transferred as a group to an opaque cylindrical holding arena (30 cm diameter), located within the center of the arena. After 10 minutes of acclimation the container was lifted, and fish were allowed to swim freely for 15 min, filmed from above by stationary GoPro Hero 4 (GoPro, California, USA) at 30 frames per second. To avoid contamination, the control group was always released into the arena first, followed by the mixed group and the oil only group last. At the end of each day the arena was drained, rinsed and allowed to dry before being refilled one h before the next assay began. All open field assays were completed within two h between 9 AM and 12 PM, with no more than 3 groups assessed per day. Owing to logistical constraints, water changes were not done between groups. Importantly, however, we believe that the effects of any olfactory cues accumulating across trials are minimal or non-existent. Firstly, the total time between the first and last trial done each day was relatively short (less than two h), with times that fish were in the arena being equal to only 60 min maximum before the last trial. Secondly, fish were fasted and so no feces were left in the tanks after testing that could potentially affect fish behavior. Additionally, there was no difference in the behavior of the untreated fish in the control and LM groups, indicating that even if there were residual cues, chemical signals, and scents left behind by the previous group, it was not enough to affect the fish in the latter groups. Finally, there were several days in which only two trials were performed, with either a mixed or oil treatment group being performed either first or second during the day. Among these groups, there was no effect of trial order on the behavior of fish in any treatment (linear mixed effect models, p > 0.50 for all behaviors in all cases).

Videos were analyzed using Ethovison (Version 10; Noldus, Wageningen, Netherlands), which was able to track fish based on the unique colored beads. The following variables were quantified for each fish within each shoal: (1) average speed; (2) average distance from the arena wall; and (3) average mean distance between the focal fish and all other fish within the shoal. All variables were measured continuously throughout each video but then aggregated using mean values within 5 min time bins throughout the trial (see Data Analysis, below).

This research was approved by the Institutional Animal Care and Use Committee of the university at which the research took place (reference number AUP-2015-00147) and followed to the ASAB/ABS Guidelines for the Use of Animals in Research. Of the 200 fish obtained for experimental purposes only two did not survive post anesthesia, neither of these fish were of the groups exposed to oil.

### Data analysis

All analyses were conducted using R v. 3.4.0 (R Development Core Team 2017) using the function lmer in package lme4^73^ for linear mixed effect models, MuMIn 1.9.13 for determining model effect sizes (marginal and conditional R^2^)^74^; (http://CRAN.R-project.org/package=MuMIn). All plots were created using the package ggplot2^75^. An initial set of linear mixed effects models (LMEs) were fitted using restricted maximum likelihood estimation, with a separate model using each of speed, distance from arena wall, and mean neighbor distance as the response variable. The models also included fish body mass and group exposure treatment (control, HO, HM, LO, LM) as categorical fixed effects. To account for any shifts in behavior over the course of each trial, videos were split into 3 different bins (5 min each) so that time over the course of the study could be included as a fixed effect. Individual nested within group was included as a random effect. To compare behaviors between exposed and unexposed fish within the mixed groups, and to compare unexposed fish within mixed groups to the control fish, a second set of LMEs were constructed with a given behavioral index as the response variable, fish body mass as a continuous fixed effect, and individual treatment (control, low exposed, low unexposed, high exposed, high unexposed) as a categorical fixed effect, and individual nested with group as a random effect. For all models, interactions between treatment and time were included, but dropped when not significant and the models re-run. Model assumptions were verified by visual examination of residual-fit plots. Significance testing (α = 0.05) was employed to provide some indication of the strength of evidence for observed patterns, along with model R^2^ values. This included marginal R^2^ (R^2^_M_) and conditional R^2^ (R^2^_C_) which indicate the variance explained by fixed factors and by both fixed and random factors, respectively^76^.

## Supplementary information

Supplemental Information

## References

[1] Chapman BB, Ward AJW, Krause J (2008). Schooling and learning: early social environment predicts social learning ability in the guppy, *Poecilia reticulata*. Animal Behaviour.

[2] Wark Abigail R., Greenwood Anna K., Taylor Elspeth M., Yoshida Kohta, Peichel Catherine L. (2011). Heritable Differences in Schooling Behavior among Threespine Stickleback Populations Revealed by a Novel Assay. PLoS ONE.

[3] Krause, J. & Ruxton, G. D. Living in groups. *Living in groups* (2002).

[4] Ward, A. & Webster, M. Sociality: The Behaviour of Group-Living Animals (2016).

[5] Brown C, Irving E (2014). Individual personality traits influence group exploration in a feral guppy population. Behavioral Ecology.

[6] Song Z, Boenke MC, Rodd FH (2011). Interpopulation Differences in Shoaling Behaviour in Guppies (*Poecilia reticulata*): Roles of Social Environment and Population Origin. Ethology.

[7] Killen SS, Marras S, Steffensen JF, Mckenzie DJ (2012). Aerobic capacity influences the spatial position of individuals within fish schools. Proceedings of the Royal Society B: Biological Sciences.

[8] Marras S (2015). Fish swimming in schools save energy regardless of their spatial position. Behavioral Ecology and Sociobiology.

[9] Weihs D (1973). Hydromechanics of Fish Schooling. Nature.

[10] Kimbell HS, Morrell LJ (2015). Turbidity in fluences individual and group level responses to predation in guppies, *Poecilia reticulata*. Animal Behaviour.

[11] Ryan MR, Killen SS, Gregory RS, Snelgrove PVR (2012). Predators and distance between habitat patches modify gap crossing behaviour of juvenile Atlantic cod (*Gadus morhua*, L. 1758). Journal of Experimental Marine Biology and Ecology.

[12] Tien JH, Levin SA, Rubenstein DI (2004). Dynamics of fish schools: identifying key decision rules. Evolutionary Ecology Research.

[13] Domenici Paolo, Steffensen John F., Marras Stefano (2017). The effect of hypoxia on fish schooling. Philosophical Transactions of the Royal Society B: Biological Sciences.

[14] Killen, S. S., Marras, S., Nadler, L., Domenici, P. & Killen, S. S. The role of physiological traits in assortment among and within fish shoals. *Philosophical Transactions of the Royal Society B: Biological Sciences* **372** (2017). 10.1098/rstb.2016.0233 PMC5498295 28673911

[15] Nadler LE, Killen SS, Mccormick MI, Watson S-A, Munday PL (2016). Effect of elevated carbon dioxide on shoal familiarity and metabolism in a coral reef fish. Conservation. Physiology.

[16] Herbert-Read, J. E., Kremer, L., Bruintjes, R., Radford, A. N. & Ioannou, C. C. Anthropogenic noise pollution from pile-driving disrupts the structure and dynamics of fish shoals. *Proceedings of the Royal Society B* **284** (2017). 10.1098/rspb.2017.1627 PMC5627215 28954915

[17] Scott GR, Sloman KA (2004). The effects of environmental pollutants on complex fish behaviour: integrating behavioural and physiological indicators of toxicity. Aquatic Toxicology.

[18] Ward AJW, Duff AJ, Horsfall JS, Currie S (2008). Scents and scents-ability: pollution disrupts chemical social recognition and shoaling in fish. Proceedings of the Royal Society B.

[19] Marras Stefano, Domenici Paolo (2013). Schooling Fish Under Attack Are Not All Equal: Some Lead, Others Follow. PLoS ONE.

[20] Reebs SG (2000). Can a minority of informed leaders determine the foraging movements of a fish shoal?. Animal Behaviour.

[21] Modlmeier AP, Keiser CN, Watters JV, Sih A, Pruitt JN (2014). The keystone individual concept: an ecological and evolutionary overview. Animal Behaviour.

[22] Keiser, C. N. & Pruitt, J. N. Personality composition is more important than group size in determining collective foraging behaviour in the wild. *Proceedings of the Royal Society B* **281** (2014). 10.1098/rspb.2014.1424 PMC4213636 25320170

[23] Douben, P. E. T. PAHs: An Ecotoxicological (2003).

[24] Readman JW (2002). Petroleum and PAH contamination of the Black Sea. Marine Pollution Bulletin.

[25] Crone TJ, Tolstoy M (2010). Magnitude of the 2010 Gulf of Mexico Oil Leak. Science.

[26] McNutt MK (2012). Review of flow rate estimates of the Deepwater Horizon oil spill. Proc Natl Acad Sci USA.

[27] Edmunds RC (2015). Corresponding morphological and molecular indicators of crude oil toxicity to the developing hearts of mahi mahi. Scientific Reports.

[28] Esbaugh AJ (2016). The effects of weathering and chemical dispersion on Deepwater Horizon crude oil toxicity to mahi-mahi (*Coryphaena hippurus*) early life stages. Science of the Total Environment.

[29] Khursigara AJ, Perrichon P, Martinez Bautista N, Burggren WW, Esbaugh AJ (2017). Cardiac function and survival are affected by crude oil in larval red drum, *Sciaenops ocellatus*. Sci Total Environ.

[30] Incardona JP (2014). Deepwater Horizon crude oil impacts the developing hearts of large predatory pelagic fish. Proceedings of the National Academy of Sciences.

[31] Stieglitz JD (2016). A novel system for embryo-larval toxicity testing of pelagic fish: Applications for impact assessment of Deepwater Horizon crude oil. Chemosphere.

[32] Brette F (2014). Excitation-Contraction Coupling in Fish. Science.

[33] Brette, F. *et al*. A Novel Cardiotoxic Mechanism for a Pervasive Global Pollutant. *Scientific Reports*, 1–9, 10.1038/srep41476 (2017). 10.1038/srep41476 PMC5282528 28139666

[34] Nelson D (2016). Effects of crude oil on *in situ* cardiac function in young adult mahi–mahi (*Coryphaena hippurus*). Aquatic Toxicology.

[35] Nelson D (2017). Cardio-respiratory function during exercise in the cobia, *Rachycentron canadum*: The impact of crude oil exposure. Comparative Biochemistry and Physiology Part - C: Toxicology and Pharmacology.

[36] Cox GK (2017). Oil Exposure Impairs *in Situ* Cardiac Function in Response to β-Adrenergic Stimulation in Cobia (*Rachycentron canadum*). Environmental Science and Technology.

[37] Claireaux G (2004). Influence of oil exposure on the physiology and ecology of the common sole *Solea solea*: Experimental and field approaches. Aquat. Living Resour..

[38] Claireaux G (2013). Effects of oil exposure and dispersant use upon environmental adaptation performance and fitness in the European sea bass, *Dicentrarchus labrax*. Aquat Toxicol.

[39] Johansen JL, Esbaugh AJ (2017). Sustained impairment of respiratory function and swim performance following acute oil exposure in a coastal marine fish. Aquatic Toxicology.

[40] Mager EM (2014). Acute embryonic or juvenile exposure to Deepwater Horizon crude oil impairs the swimming performance of mahi-mahi (*Coryphaena hippurus*). Environ Sci Technol.

[41] Davison W, Franklin CE, Mckenzie JC, Dougan MCR (1992). The effects of acute exposure to the water soluble fraction of diesel fuel oil on survival and metabolic rate in antartic fish (*Pagothenia Borchgrevinki*). Comparative Biochemistry and Physiology.

[42] Klinger DH (2015). Exposure to Deepwater Horizon weathered crude oil increases routine metabolic demand in chub mackerel, *Scomber japonicus*. Marine Pollution Bulletin.

[43] Ankley GT (2010). Adverse outcome pathways: A conceptual framework to support ecotoxicology research and risk assessment. Environmental Toxicology and Chemistry.

[44] Réale D, Reader SM, Sol D, McDougall PT, Dingemanse NJ (2007). Integrating animal temperament within ecology and evolution. Biological Reviews.

[45] Jolles JW, Boogert NJ, Sridhar VH, Couzin ID, Manica A (2017). Consistent Individual Differences Drive Collective Behavior and Group Functioning of Schooling Fish. Current Biology.

[46] Killen SS (2016). The relationship between metabolic rate and sociability is altered by food deprivation. Functional Ecology.

[47] Johansen Jacob L., Allan Bridie J. M., Rummer Jodie L., Esbaugh Andrew J. (2017). Oil exposure disrupts early life-history stages of coral reef fishes via behavioural impairments. Nature Ecology & Evolution.

[48] Kowalko JE (2013). Loss of schooling behavior in cavefish through sight-dependent and sight-independent mechanisms. Current Biology.

[49] Rahn AK (2018). Parasitic infection of the eye lens affects shoaling preferences in three-spined stickleback. Biological Journal of the Linnean Society.

[50] Ward AJW, Axford S, Krause J (2002). Mixed-species shoaling in fish: The sensory mechanisms and costs of shoal choice. Behavioral Ecology and Sociobiology.

[51] Faucher K, Parmentier E, Becco C, Vandewalle N, Vandewalle P (2010). Fish lateral system is required for accurate control of shoaling behaviour. Animal Behaviour.

[52] Ward AJW, Sumpter DJT, Couzin ID, Hart PJB, Krause J (2008). Quorum decision-making facilitates information transfer in fish shoals. Proceedings of the National Academy of Sciences.

[53] Ruxton GD, Hall SJ, Gurney WSC (2018). Attraction Toward Feeding Conspecifics when Food Patches are Exhaustible. The American Naturalist.

[54] Killen SS, Marras S, Mckenzie DJ (2011). Fuel, fasting, fear: Routine metabolic rate and food deprivation exert synergistic effects on risk-taking in individual juvenile European sea bass. Journal of Animal Ecology.

[55] Burns ALJ, Herbert-Read JE, Morrell LJ, Ward AJW (2012). Consistency of Leadership in Shoals of Mosquitofish (*Gambusia holbrooki*) in Novel and in Familiar Environments. PLoS One.

[56] Mclean S, Persson A, Norin T, Killen SS (2018). Metabolic Costs of Feeding Predictively Alter the Spatial Distribution of Individuals in Fish Schools Report Metabolic Costs of Feeding Predictively Alter the Spatial Distribution of Individuals in Fish Schools. Current Biology.

[57] Laskowski, K. L. & Pruitt, J. N. Evidence of social niche construction: persistent and repeated social interactions generate stronger personalities in a social spider. *Proceedings of the Royal Society B* **281** (2014). 10.1098/rspb.2013.3166 PMC3996602 24671972

[58] Ioannou CC, Singh M, Couzin ID (2015). Potential Leaders Trade Off Goal-Oriented and Socially Oriented Behavior in Mobile Animal Groups. The American Naturalist.

[59] Faria JJ (2010). A novel method for investigating the collective behaviour of fish: introducing ‘Robofish’. Behavioral Ecology and Sociobiology.

[60] Conrad JL, Weinersmith KL, Brodin T, Saltz JB, Sih A (2011). Behavioural syndromes in fishes: a review with implications for ecology and fisheries management. J Fish Biol.

[61] Rowsey, L. E., Johansen, J. L., Khursigara, A. J. & Esbaugh, A. J. Oil exposure impairs predator-prey dynamics in larval red drum (*Sciaenops ocellatus*). *Marine and Freshwater Research* (2019).

[62] Pruitt JN, Keiser CN (2014). The personality types of key catalytic individuals shape colonies’ collective behaviour and success. Animal Behaviour.

[63] Jones KA, Croft DP, Ramnarine IW, Godin J-GJ (2009). Size-Assortative Shoaling in the Guppy (*Poecilia reticulata*): The Role of Active Choice. Ethology.

[64] Buskey, E. J., White, H. J. & Esbaugh, A. J. Impacts of Oil Spills on Marine Life in the Gulf of Mexico. *Oceanography* (2016).

[65] Collier TK (2014). Effects on Fish of Polycyclic Aromatic Exposures. *Fish*. Physiology: Organic Chemical Toxicology of Fishes.

[66] Pan YK, Khursigara AJ, Johansen JL, Esbaugh AJ (2018). The effects of oil induced respiratory impairment on two indices of hypoxia tolerance in Atlantic croaker (*Micropogonias undulatus*). Chemosphere.

[67] Xu EG (2017). Larval Red Drum (*Sciaenops ocellatus*) Sublethal Exposure to Weathered Deepwater Horizon Crude Oil: Developmental and Transcriptomic Consequences. Environ Sci Technol.

[68] Xu, E. G. *et al*. Time- and Oil-Dependent Transcriptomic and Physiological Responses to Deepwater Horizon Oil in Mahi-Mahi (*Coryphaena hippurus*) Embryos and Larvae. *Environmental Science & Technology*, acs.est.6b02205 (2016). 10.1021/acs.est.6b02205 27348429

[69] Diercks AR (2010). Characterization of subsurface polycyclic aromatic hydrocarbons at the Deepwater Horizon site. Geophysical Research Letters.

[70] Killen SS, Adriaenssens B, Marras S, Claireaux G, Cooke SJ (2016). Context dependency of trait repeatability and its relevance for management and conservation of fish populations. Conserv Physiol.

[71] Mckenzie DJ, Belaõ TC, Killen SS, Rantin FT (2016). To boldly gulp: standard metabolic rate and boldness have context-dependent influences on risktaking to breathe air in a catfish. Journal of Experimental Biology.

[72] Bejarano AC, Levine E, Mearns AJ (2013). Effectiveness and potential ecological effects of offshore surface dispersant use during the Deepwater Horizon oil spill: a retrospective analysis of monitoring data. Environ Monit Assess.

[73] Bates, D. *et al*. Package ‘lme4’. *In R Package Version 11*–*10*, 2016 (2016).

[74] Barton K (2015). MuMIn: Multi-Model Inference. In R Package Version.

[75] Wickham, H., Chang, W. & Package, W. M. H. Package ‘ggplot2’ (2013).

[76] Nakagawa S, Schielzeth H (2010). Repeatability for Gaussian and non-Gaussian data: a practical guide for biologists. Biological Reviews.

