# Supplementary material for: Oil exposure alters social group cohesion in fish
Source: Sci Rep. 2019 Sep 18;9:13520. doi: 10.1038/s41598-019-49994-1 (PMC6751191; doi:10.1038/s41598-019-49994-1)
Supplement: Supplementary file 1 — Supplemental Information [file 41598_2019_49994_MOESM1_ESM.docx]

**Oil exposure alters social group cohesion in fish**

Tiffany Armstrong^1 #^, Alexis J. Khursigara^2 #^, Shaun S. Killen^1^, Hannah Fearnley^1^, Kevin J. Parsons^1^, Andrew J. Esbaugh^2^

^1^ University of Glasgow, Institute of Biodiversity, Animal Health and Comparative Medicine, Glasgow UK G12 8QQ

^2^ University of Texas at Austin, University of Texas Marine Science Institute, Port Aransas, Texas, USA 78373

^#^ These authors contributed equally to the study.

**Table S1**. Tukey HSD pairwise comparisons for the treatment levels for the models presented in Table 1 in the main text. HO = high oil; LO = low oil; HM = high mixed; LM = low mixed.

|  |  | **Difference** | **Adjusted P** |
| --- | --- | --- | --- |
| **Speed (cm s^-1^)** | |  |  |
| HO-control |  | -1.684 | <0.0001 |
| LO-control |  | -1.053 | 0.037 |
| HM-control |  | 0.074 | 0.999 |
| LM-control |  | -0.373 | 0.851 |
| LO-HO |  | 0.631 | 0.431 |
| HM-HO |  | 1.758 | <0.0001 |
| LM-HO |  | 1.311 | 0.004 |
| HM-LO |  | 1.127 | 0.019 |
| LM-LO |  | 0.68 | 0.423 |
| LM-HM |  | -0.447 | 0.74 |
|  |  |  |  |
| **Distance to arena edge (cm)** | | |  |
| HO-control |  | -1.085 | 0.489 |
| LO-control |  | 0.383 | 0.985 |
| HM-control |  | -3.266 | <0.0001 |
| LM-control |  | -1.272 | 0.404 |
| LO-HO |  | 1.468 | 0.258 |
| HM-HO |  | -2.181 | 0.01 |
| LM-HO |  | -0.187 | 0.999 |
| HM-LO |  | -3.649 | <0.0001 |
| LM-LO |  | -1.655 | 0.209 |
| LM-HM |  | 1.994 | 0.046 |
|  |  |  |  |
| **Mean distance to neighbors (cm)** | | |  |
| HO-control |  | 0.186 | <0.0001 |
| LO-control |  | 0.035 | 0.348 |
| HM-control |  | 0.155 | <0.0001 |
| LM-control |  | 0.027 | 0.611 |
| LO-HO |  | -0.152 | <0.0001 |
| HM-HO |  | -0.031 | 0.37 |
| LM-HO |  | -0.159 | <0.0001 |
| HM-LO |  | 0.12 | <0.0001 |
| LM-LO |  | -0.008 | 0.995 |
| LM-HM |  | -0.128 | <0.0001 |

**TABLE S2.** Results of linear mixed effects models comparing oil exposed and unexposed within mixed groups, and to fish in control groups in which no fish were oil exposed. For the fixed effect “individual treatment”, control groups are the reference level. Models included individual nested within group as a random effect.

|  | **Estimate** | **SEM** | **df** | **t** | **p** | **R^2^_m_** | **R^2^_c_** |
| --- | --- | --- | --- | --- | --- | --- | --- |
| **Speed (cm s^-1^)** |  |  |  |  |  |  |  |
| intercept | 0.016 | 1.174 | 89.93 | 0.014 | 0.989 | 0.214 | 0.84 |
| mass | 0.184 | 0.048 | 86.09 | 3.825 | 0.0002 |  |  |
| time | 0.201 | 0.017 | 177.06 | 11.998 | < 0.0001 |  |  |
| individual treatment |  |  |  |  |  |  |  |
| high mix exposed | -0.042 | 0.874 | 87.61 | -0.048 | 0.962 |  |  |
| high mix unexposed | 0.151 | 0.601 | 87.08 | 0.251 | 0.802 |  |  |
| low mix exposed | -0.371 | 1.022 | 87.75 | -0.363 | 0.718 |  |  |
| low mix unexposed | -0.304 | 0.674 | 85.75 | -0.505 | 0.615 |  |  |
|  |  |  |  |  |  |  |  |
| **Distance to arena edge (cm)** |  |  |  |  |  |  |  |
| intercept | 16.161 | 1.985 | 91.869 | 8.14 | < 0.0001 | 0.119 | 0.696 |
| mass | 0.0099 | 0.081 | 85.515 | 0.124 | 0.902 |  |  |
| time | -0.149 | 0.038 | 177.727 | -3.872 | 0.0002 |  |  |
| individual treatment |  |  |  |  |  |  |  |
| high mix exposed | -2.723 | 1.471 | 87.836 | -1.851 | 0.068 |  |  |
| high mix unexposed | -3.278 | 1.01 | 87.022 | -3.246 | 0.001 |  |  |
| low mix exposed | 0.809 | 1.712 | 84.997 | 0.473 | 0.638 |  |  |
| low mix unexposed | -1.927 | 1.129 | 84.993 | -1.706 | 0.092 |  |  |
|  |  |  |  |  |  |  |  |
| **Mean distance to neighbors (cm)** | |  |  |  |  |  |  |
| intercept | 1.048 | 0.057 | 89.64 | 18.343 | < 0.0001 | 0.252 | 0.835 |
| mass | 0.002 | 0.023 | 85.97 | 0.887 | 0.378 |  |  |
| time | 0.0004 | 0.0009 | 177.1 | 0.501 | 0.617 |  |  |
| individual treatment |  |  |  |  |  |  |  |
| high mix exposed | 0.164 | 0.043 | 87.6 | 3.852 | 0.000223 |  |  |
| high mix unexposed | 0.148 | 0.029 | 87.03 | 5.063 | < 0.0001 |  |  |
| low mix exposed | 0.4118 | 0.051 | 85.61 | 0.842 | 0.402 |  |  |
| low mix unexposed | 0.233 | 0.033 | 85.6 | 0.711 | 0.479 |  |  |

**Table S3.** Tukey HSD pairwise comparisons for the treatment levels for the models presented in Table S2. HO = high oil; LO = low oil; HM = high mixed; LM = low mixed.

|  |  | **Difference** | **Adjusted P** |
| --- | --- | --- | --- |
| **Speed (cm s^-1^)** | |  |  |
|  | HM(exposed)-control | 0.054 | 0.999 |
|  | HM(unexposed)-control | 0.09 | 0.999 |
|  | LM(exposed)-control | -0.359 | 0.978 |
|  | LM(unexposed)-control | -0.337 | 0.926 |
|  | HM(unexposed)-HM(exposed) | 0.037 | 0.999 |
|  | LM(exposed)-HM(exposed) | -0.412 | 0.982 |
|  | LM(unexposed)-HM(exposed) | -0.391 | 0.964 |
|  | LM(exposed)-HM(unexposed) | -0.449 | 0.956 |
|  | LM(unexposed)-HM(unexposed) | -0.427 | 0.863 |
|  | LM(unexposed)-LM(exposed) | 0.022 | 0.999 |
|  |  |  |  |
| **Distance to arena edge (cm)** | |  |  |
|  | HM(exposed)-control | -3.038 | 0.016 |
|  | HM(unexposed)-control | -3.338 | < 0.0001 |
|  | LM(exposed)-control | 0.774 | 0.957 |
|  | LM(unexposed)-control | -1.937 | 0.066 |
|  | HM(unexposed)-HM(exposed) | -0.3 | 0.998 |
|  | LM(exposed)-HM(exposed) | 3.812 | 0.037 |
|  | LM(unexposed)-HM(exposed) | 1.101 | 0.829 |
|  | LM(exposed)-HM(unexposed) | 4.112 | 0.003 |
|  | LM(unexposed)-HM(unexposed) | 1.402 | 0.364 |
|  | LM(unexposed)-LM(exposed) | -2.711 | 0.146 |
|  |  |  |  |
| **Mean distance to neighbors (cm)** | |  |  |
|  | HM(exposed)-control | 0.172 | < 0.0001 |
|  | HM(unexposed)-control | 0.15 | < 0.0001 |
|  | LM(exposed)-control | 0.042 | 0.641 |
|  | LM(unexposed)-control | 0.023 | 0.776 |
|  | HM(unexposed)-HM(exposed) | -0.022 | 0.933 |
|  | LM(exposed)-HM(exposed) | -0.129 | 0.0048 |
|  | LM(unexposed)-HM(exposed) | -0.148 | < 0.0001 |
|  | LM(exposed)-HM(unexposed) | -0.107 | 0.006 |
|  | LM(unexposed)-HM(unexposed) | -0.126 | < 0.0001 |
|  | LM(unexposed)-LM(exposed) | -0.019 | 0.978 |
